# Influence of Feed Composition on the Separation Factor during Nanofiltration of Organic Acids

**DOI:** 10.3390/membranes14080166

**Published:** 2024-07-28

**Authors:** Gustavo Tottoli, Sylvain Galier, Hélène Roux-de Balmann

**Affiliations:** Laboratoire de Génie Chimique, Université de Toulouse, CNRS, INPT, UPS, 118 Route de Narbonne, CEDEX 4, 31062 Toulouse, France; gustavo.machado-tottoli-ferreira@univ-tlse3.fr (G.T.); sylvain.galier@univ-tlse3.fr (S.G.)

**Keywords:** nanofiltration, organic acids, separation factor, feed concentration, solute proportions

## Abstract

In this study, nanofiltration experiments using synthetic solutions containing acetate, butyrate, and lactate are carried out to assess the impact of the feed composition, i.e., feed concentration and feed proportions, on the separation factor of couples of solutes in binary and ternary solutions. In binary solutions, no influence of the solute proportions in the feed was pointed out, whatever the couple of solutes. The separation factor of acetate/butyrate and acetate/lactate was found to decrease with increasing feed concentration, while that of lactate/butyrate remained constant. The separation factors of acetate/lactate and lactate/butyrate were identical in ternary solutions compared to binary ones, showing no impact of the addition of the third solute. In ternary solutions, the presence of lactate decreased the separation factor of acetate/butyrate, but this decrease was not influenced by the proportion of lactate.

## 1. Introduction

Short-chained carboxylic organic acids like acetate, butyrate, and lactate have garnered significant attention in recent years as key building blocks for a wide range of industrial applications [1]. These organic compounds are valuable precursors for the synthesis of biofuels, biopolymers, and various chemical products [2]. Acetate and butyrate, in particular, are naturally occurring volatile fatty acid (VFA) products of biological fermentation processes [3]. While lactate is not a VFA, it can also be obtained as a by-product of fermentation processes with mixed microbial cultures [4].

Membrane-based separation techniques, such as nanofiltration (NF), have emerged as promising technologies for organic acid (OA) recovery [5]. Nanofiltration is a separation technique that relies on a pressure gradient to perform liquid-phase separation. It was established that the retention of solutes and so the selectivity in nanofiltration are the result of a combination of size (steric hindrance), charge (electrostatic interaction) effects, and more recently, dielectric exclusion [6,7]. Short-chain carboxylic acids like acetate, butyrate, and lactate are weak acids. Since fermentation processes are mostly operated at a pH higher than the OA’s pKa, they are mostly produced in their dissociated form as anions [8].

Numerous studies have investigated the retention of solutes during nanofiltration of mixtures of various complexity, such as synthetic solutions as well as fermentation broths or hydrolysates, for instance [9,10,11]. However, very few have been dedicated to investigating the selectivity of NF expressed by the separation factor (*SF*) and especially the effects of varying the feed composition, i.e., solute concentration and proportions, on the *SF*. Moreover, the conclusions sometimes differ regarding these effects. For example, it was observed that the separation factor of succinate from acetate at pH 7 decreases with increasing feed concentration using an NF45 membrane [12]. In a different paper, no significant effect of concentration on the retention of acetate, butyrate, and propionate at pH 8.5 was reported with the dNF40 membrane [13]. Given that the separation factor is defined as a relationship between retentions, this means that the *SF* was independent of concentration.

In other papers, the effect of varying the feed concentration on the separation factor depends on the membrane. For instance, it was reported that for a lignocellulosic hydrolysate, the feed concentration influenced the retention of xylose and glucose with a membrane (NF270), while no influence was observed with another one (NF90) [11]. A different paper reported similar findings comparing two different membranes (ES10 and NF270) [14]. The authors evaluated the retention of formic and acetic acids at pH 7 and observed that the retention and, thus, the separation factor were influenced by the feed concentration for only one of the two membranes (NF270).

Furthermore, the influence of the feed proportions of xylose and glucose on solute flux was studied with a Desal-5 DL membrane [15]. From the data reported in this paper, the separation factor can be calculated and found to be approximately constant for the different xylose-to-glucose proportions. In a recent study, it was observed that, with a NF45 membrane and at pH 8, the proportions of acetate, propionate, and butyrate in the permeate were not dependent on the total feed concentration but only on the solute proportions in the feed [16]. Based on the data provided in this paper, one can calculate the *SF*, which is the ratio between the solute proportion in the permeate and in the feed. It was shown that this ratio does not depend on the feed concentration, the solute proportion in the feed, or whether they are present in binary or ternary solutions.

Thus, the purpose of this study is to conduct an experimental evaluation of the effect of varying the feed composition, i.e., concentration and solute proportions, on the retention and separation factor of organic acids, specifically acetate, butyrate, and lactate, in nanofiltration. Separation factors obtained in binary solutions are compared to those obtained with ternary mixtures to point out, if any, the influence of a third solute. Findings from this study are also discussed in light of previous ones in order to draw possible general conclusions.

## 2. Materials and Methods

### 2.1. Membrane and Chemicals

This study was conducted with a commercially available nanofiltration membrane, NF270, supplied by Dow FilmTec (Midland, MI, USA). The membrane is composed of a polymeric thin-film composite material with a nominal molecular weight cut-off value of 200–300 Da [17] and a negatively charged surface at pH values above its isoelectric point of 3.5 [18]. To ensure that no membrane degradation or aging occurred, the pure water permeability was consistently checked prior to and following each experiment. In addition, the retention of a neutral reference molecule (glucose molecular weight: 180 g·mol^−1^) was frequently checked. The variation of the pure water permeability did not exceed 12%, and the variation in retention of the reference molecule did not exceed 6%. There were no signs of deterioration, degradation, or alterations in the membrane’s properties throughout the ensemble of experiments.

Sodium acetate (NaAc) and sodium butyrate (NaBu) were obtained from Alfa Aesar (Haverhill, MA, USA). Sodium lactate (NaLa) was obtained from Acros Organics (Geel, Belgium). Stock solutions of NaAc, NaBu, and NaLa were prepared at specified concentrations using ultrapure water as the solvent, and the pH was 8. No pH adjustment was necessary. Table 1 presents the chemical characteristics of acetate, butyrate, and lactate.

### 2.2. Experimental Set-Up and Procedure

The experiments were carried out on a stirred cylindrical dead-end filtration cell made of 316 stainless steel. The operating conditions are presented in Table 2.

The experimental procedure was conducted as previously described by Zhu et al. (2020) [16]. Table 3 summarizes the conditions for each experiment. Experiments S4, S7, S10, and S13, corresponding to equimolar mixtures of organic acids, were conducted at 150, 300, and 500 mM. Experiments 14 and 15, corresponding to different proportions of OA in ternary solutions, were conducted at 150 mM. All other experiments were conducted at 150 and 300 mM.

### 2.3. Analytical Methods

The concentrations of acetate, butyrate, and lactate in the samples were determined using High-Performance Liquid Chromatography (HPLC) (Jasco LC Net II/ADC, Jasco, Tokyo, Japan). The system was equipped with an Aminex HPX-87H column (BioRad, Hercules, CA, USA) and a UV detector (UV-2077 plus, Easton, MD, USA) set at a wavelength of 210 nm. The column temperature was maintained at 65 °C. The mobile phase consisted of 0.5 g·L^−1^ sulfuric acid with a flow rate of 0.6 mL·min^−1^. The injection volume for the samples was 10 µL. The concentrations of organic acids were calculated based on a calibration curve previously established.

### 2.4. Data Treatment

The filtration flux *J_v_* (m·s^−1^) is calculated by Equation (1).

(1) Jv=Vpt×Sm

where *J_v_* is defined as the total volume *V_p_* (m^3^) permeated through the membrane within a time *t* (s) per unit of the membrane area *S_m_* (m^2^). The membrane’s water permeability *L_p_*_0_ (m·s^−1^·Pa^−1^) is calculated by Equation (2).

(2) $$L_{p0}=\frac{J_{v}}{∆P}$$

where *L_p_*_0_ is defined as the filtration flux of ultrapure water *J_v_* permeated through the membrane under a certain pressure Δ*P* (Pa). The retention of a solute *A*, *R_A_*, is calculated by Equation (3).

(3) RA=1−CACA0

where *C_A_*_0_ (mol·m^−3^) and *C_A_* are the solute concentrations in the feed and in the permeate. The retention versus filtration flux curves were fitted using a model as previously described by Zhu et al. [16]. Finally, the separation factor between two solutes *A* and *B* is calculated by Equation (4).

(4) SF=CACA0CBCB0=1−RA1−RB

The *SF* can be seen as an enrichment factor. If *SF* = 1, there is no difference between the retentions of the two solutes, and the composition is the same in the permeate compared to the feed. If *SF* > 1, solute *A* is enriched in the permeate relative to solute *B* compared to the feed. The larger the value of *SF*, the more pronounced the enrichment of one solute over the other.

The statistical analysis of the data was performed using the software OriginPro 2023 (OriginLab Corporation, Northampton, MA, USA). An analysis of variance (ANOVA) was conducted to determine whether there were significant differences between groups of data obtained for different experimental conditions. The threshold for statistical significance is above the significance level of 0.05 [19].

## 3. Results

### 3.1. Membrane Characterization

Prior to evaluating the retention of organic acids in synthetic solutions, the NF270 membrane is characterized in terms of its pure water permeability and retention of a neutral reference molecule. The membrane’s pure water permeability (*L_p_*_0_) is calculated by measuring the water flux under different applied pressures, as described by Equation (2). The slope of the linear fit corresponds to the *L_p_*_0_ and is 4.7 × 10^−11^ m·s^−1^·Pa^−1^. The retention for a neutral reference molecule, in this case, glucose, versus the applied pressure plateaus at 73%.

### 3.2. Single Solutions

**Figure 1:**
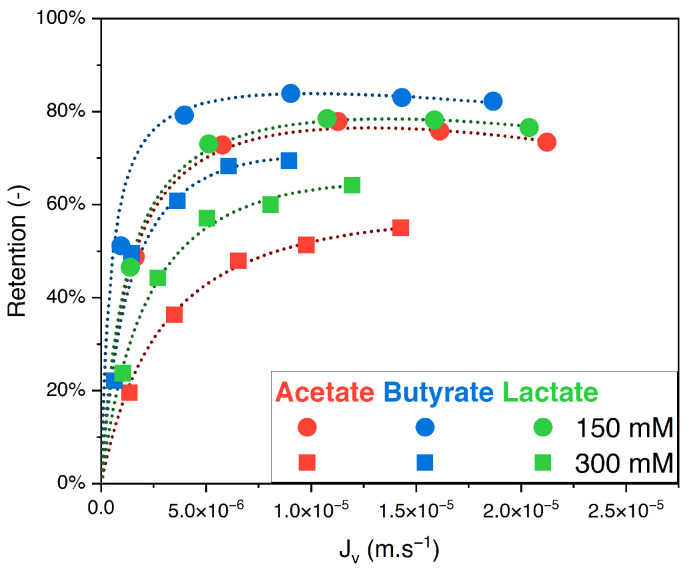
Retention vs. filtration flux of organic acids in single solution at 150 mM and 300 mM using an NF270 membrane.

The retention of acetate, butyrate, and lactate in single solutions as a function of the filtration flux at 150 and 300 mM feed concentrations and pH 8 is presented in Figure 1.

For both feed concentrations, the retention order of the organic acids is, from highest to lowest: butyrate, lactate, and acetate. Moreover, the retention decreases with the initial concentration in the feed solution. At the highest pressure (20 bar) and an initial concentration of 150 mM, the retentions were, respectively, 82% for butyrate, 77% for lactate, and 73% for acetate. When the concentration increased to 300 mM, the retentions were, respectively, 69% for butyrate, 64% for lactate, and 55% for acetate.

At pH 8, acetate, butyrate, and lactate are fully dissociated and negatively charged, as is the NF270 membrane [20]. Being so, retention is subjected to charge screening, which refers to the reduction in electrostatic interactions between the solute and the membrane as total concentration increases, leading to decreased retention [21,22,23]. Moreover, as expected, the filtration flux for a given organic acid decreases with increasing initial concentrations [11,24].

Based on the molecular weight and the steric exclusion, one might expect the retention of butyrate to be similar to that of lactate and higher than that of acetate. However, the results show that the retention of lactate is lower than that of butyrate. Two possible hypotheses could explain this difference, i.e., the first is the different molecular conformation between lactate and butyrate. Indeed, despite their similar molecular weights, butyrate has a more linear conformation, whereas lactate has a more compact conformation. The difference in their spatial arrangements could influence the retention. A similar observation was made in the work of Laufenberg et al. (1996) [25]. The authors worked with reverse osmosis membranes and noted that, in single solutions, both secondary isomers of butyric and valeric acid were better retained than their primary isomers by 18 and 14%, respectively (pH was not disclosed).

The second hypothesis for the lower retention of lactate compared to butyrate is related to the influence of hydroxyl groups and hydration phenomena. Previous works in the literature correlate the hydration properties of the solutes with their retention in nanofiltration. In the work of Teychené et al. (2021) [26], the authors evaluated the variation in the retention of neutral solutes across nanofiltration membranes in the presence of electrolytes. It was found that the impact of adding an electrolyte on sugar retention was directly related to the number of hydroxyl groups present in the sugar. This suggests that the difference in the hydroxyl groups in lactate (having an “extra” OH group) compared to butyrate could play a role in the observed retention order.

### 3.3. Binary Solutions

**Figure 2:**
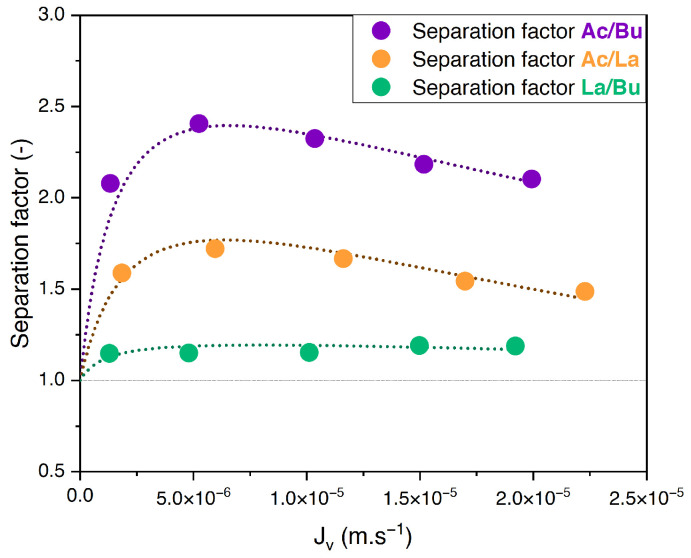
Separation factor vs. filtration flux for equimolar binary solutions of acetate, butyrate, and lactate at 150 mM using an NF270 membrane.

The separation factor of equimolar binary solutions of acetate/butyrate, acetate/lactate, and butyrate/lactate at an initial concentration of 150 mM and pH 8 versus the filtration flux is presented in Figure 2.

One can see that the separation factors of acetate/butyrate and acetate/lactate undergo slight variations with the filtration flux, increasing slightly at lower filtration fluxes and reaching a maximum value before gradually decreasing. However, they are overall maintained at an approximately constant level within this range of filtration fluxes. The separation factor of lactate/butyrate is constant throughout the range of filtration fluxes studied. The average values for the separation factors for acetate/butyrate, acetate/lactate, and lactate/butyrate are 2.16, 1.53, and 1.18, respectively.

Similar observations in the relationship between the separation factor and the filtration flux are found in the literature. Zhu et al. (2020) [16] observed that there is a threshold value for the filtration flux above which the proportions of acetate, propionate, and butyrate in the permeate are constant in a dead-end filtration cell. Consequentially, the separation factors for acetate/butyrate, acetate/propionate, or propionate/butyrate were independent of the filtration flux above said threshold value. 

#### 3.3.1. Effect of Feed Concentration

**Figure 3:**
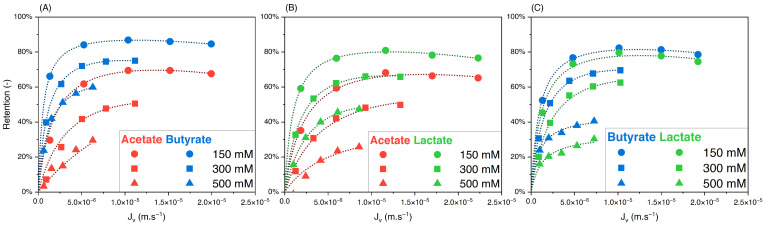
Retention vs. filtration flux of equimolar binary solutions of (**A**) acetate/butyrate, (**B**) acetate/lactate, and (**C**) butyrate/lactate at 150, 300, and 500 mM using an NF270 membrane.

**Figure 4:**
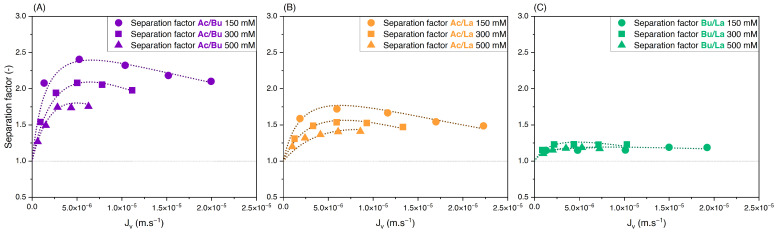
Separation factor vs. filtration flux of equimolar binary solutions of (**A**) acetate/butyrate, (**B**) acetate/lactate, and (**C**) butyrate/lactate at 150, 300, and 500 mM using an NF270 membrane.

To assess the effect of the feed concentration on the retention and separation factors of the solutes in binary solutions, equimolar solutions of acetate/butyrate, acetate/lactate, and butyrate/lactate were used at different initial concentrations of 150, 300, and 500 mM. Variations of retention versus filtration flux are presented in Figure 3A–C. The corresponding separation factors for the three binaries are presented in Figure 4A–C.

As observed with single solutions, Figure 3 shows that the retention of organic acids decreases with initial concentration due to charge screening. Based on Figure 4A, the separation factor of acetate/butyrate decreases with increasing feed concentrations, with average values of 2.16, 2.00, and 1.72, respectively, at 150, 300, and 500 mM. A similar trend is seen for the binary acetate/lactate (Figure 4B), where the average values for the separation factor are 1.53, 1.49, and 1.39 for the same concentrations. These results indicate that, in the case of acetate/butyrate and acetate/lactate binaries and at pH 8, the electrostatic repulsion (higher at lower feed concentrations) is favorable to the separation factor. The separation factor of the binary lactate/butyrate seems to be very little influenced by the initial concentration, averaging at 1.18 ± 0.04, as indicated in Figure 4C. While a statistical difference is identified by a one-way ANOVA test (*p* < 0.05), the mean values of the separation factor are quite similar, i.e., 1.17 at 150 mM, 1.21 at 300 mM, and 1.16 at 500 mM.

The work of Li et al. (2003) [9] assessed the separation of l-glutamine (l-Gln) and l-glutamate (l-Glu) from a fermentation broth by nanofiltration using a NTR7450 NF membrane. At pH 7, these solutes are both submitted to the charge exclusion mechanism. The authors observed an increase in the separation factor from 2.5 to 17 with a dilution of the fermentation broth (dilution factor from 1 to 10) and concluded that the higher concentration of the fermentation broth not only decreases the retention but also the selective separation of l-Gln from l-Glu.

Zhu et al. (2020) [16] studied the effect of feed concentration on the retention of acetate, propionate, and butyrate and the relationship between feed and permeate proportions in binary and ternary solutions in dead-end filtration using a NF45 membrane. They reported that, while the retention of these acids at pH 8 was influenced by the total feed concentration ranging from 100 to 500 mM, their proportions in the permeate were only dependent on their respective proportions in the feed. This implies that the separation factor—defined as the ratio of solute concentrations in the permeate to those in the feed—for acetate/butyrate, acetate/propionate, and propionate/butyrate was also not affected by the total feed concentration. Comparing these findings to our own, it is shown that the impact of total feed concentration on the separation factor of charged organic acids is different depending on the membrane. 

A similar trend can be pointed out for neutral solutes from the results reported by Nguyen et al. (2015) [11]. The authors evaluated the detoxification of lignocellulosic hydrolysates through nanofiltration. In their study, glucose, xylose, and arabinose, mixed with hydrolysates, were filtered using various nanofiltration membranes in a cross-flow filtration module in concentration mode, increasing the volume reduction ratio (VRR). From the data reported in this paper, the separation factor of xylose and glucose can be calculated. It is observed that using the NF270 membrane, the separation factor diminishes with an increase in the VRR from 2 to 8. On the contrary, when utilizing the NF90 membrane, retentions remained very high and constant at around 99%, irrespective of the volume reduction ratio. Then, as the retentions were unchanged, so was the separation factor.

Another study conducted by Choi et al. (2008) [14] investigated the influence of feed concentrations on the retention of organic acids using two nanofiltration membranes, ES10 and NF270, in cross-flow filtration. At pH 7 and an operating pressure of 2.8 bar, the retentions of formic and acetic acids by the ES10 membrane exceeded 90% and were not affected by increasing the feed concentration from 50 to 500 mg·L^−1^ (0.6 to 6.6 mM). The separation factor remained constant as the retentions were unchanged. On the contrary, with the NF270 membrane, the retention of formic acid decreased significantly with increasing concentration, while that of acetic acid remained constant. This represents an increase in the *SF* calculated. It is worth noting that the increase in the separation factor is an exception compared to other results, likely due to a low operating pressure and a small concentration range.

Khunnonkwao et al. (2018) [12] evaluated the separation factor of succinate over using a NF45 membrane in cross-flow (tangential) filtration. Different dilutions were used to investigate the influence of feed concentration. For the highest concentrations of 700 mM succinate and 100 mM acetate (dilution factor 1), the retentions of both solutes were low and similar, with a separation factor of approximately 1 at pressures from 2 to 20 bar. As the feed concentration decreased, the separation factor increased up to a maximum of approximately 6.5 (dilution factor 10 and filtration flux 1 × 10^−5^ m^3^·m^−2^·s^−1^).

In the study conducted by Bóna et al. (2020) [13], the authors evaluated the application of an NF membrane (dNF40) for solutions comprising acetic, butyric, and propionic acids at different concentrations and pH 8.5 in cross-flow filtration. Statistical analysis was used to ascertain the impact of the initial concentration on the retention of these acids. Based on the published results, the separation factors of acetate over butyrate and propionate over butyrate, calculated using the individual retention of acids, are approximately constant and independent of the feed concentration, ranging from 18 to 60 mM. 

Finally, Domingos et al. (2022) [27] investigated the separation of VFAs using a nanofiltration membrane (DK1812) in cross-flow filtration. The experiments were conducted in concentration mode, increasing the feed concentration over time. The authors evaluated the influence of feed concentration on the separation factor of acetic, propionic, valeric, and heptanoic acids over butyric acid. At pH 7, no significant impact of feed concentration on the separation factor was observed for total VFA concentrations up to 40 g/L. Beyond the 40 g/L threshold, the separation factor of acetate over butyrate decreased slightly from 2.0 to 1.7 at 65 g/L. It is noteworthy to mention that in concentration mode, while the overall feed concentration increases, the proportion of each solute also changes over time due to the different retentions of the solutes. Thus, the observed effects on separation factors could potentially be an interplay of both changes in overall concentration and proportions. Unfortunately, it is not possible, based on the available data, to dissociate the two effects.

Based on our results, with the NF270 membrane, the separation factor decreases with increasing feed concentration. In the literature, two possible outcomes are generally observed, i.e., the *SF* either decreases with feed concentration or remains constant depending on the membrane. For instance, the results of Nguyen et al. (2015) [11] and Choi et al. (2008) [14] indicate that the separation factor is influenced by the feed concentration with the NF270 membrane, which is in agreement with our results but not with the NF90 and ES10 ones. Interestingly, the NF90 and ES10 membranes are known to be “tighter” membranes compared to the NF270, which is known to be “looser” [28]. The same comparison can be made between our results and the ones published by Zhu et al. (2020) [16] with the NF45 membrane. Indeed, with the tighter membrane, NF45, no influence of the feed concentration was observed on the separation factor, contrary to the NF270 membrane.

#### 3.3.2. Effect of Solute Proportions

**Figure 5:**
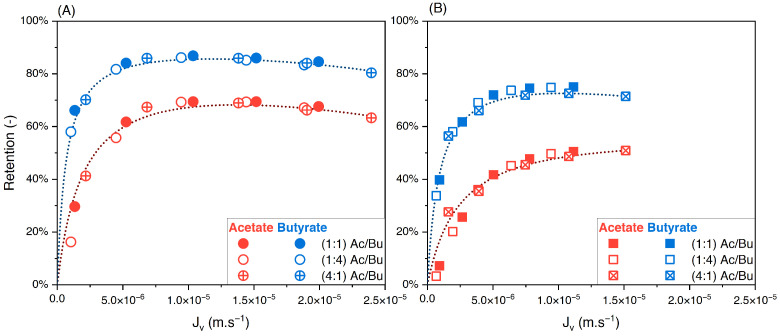
Retention of acetate and butyrate vs. filtration flux—binary solutions of acetate/butyrate with different feed proportions at (**A**) 150 and (**B**) 300 using an NF270 membrane.

To evaluate the influence of varying the feed solute proportions on the performance of nanofiltration, binary solutions of acetate/butyrate, acetate/lactate, and butyrate/lactate were studied in proportions of 1:1, 1:4, and 4:1 each at two total concentrations, i.e., 150 and 300 mM. The retentions of individual solutes in the binary acetate/butyrate as a function of the filtration flux with different feed proportions are plotted in Figure 5A,B.

**Figure 6:**
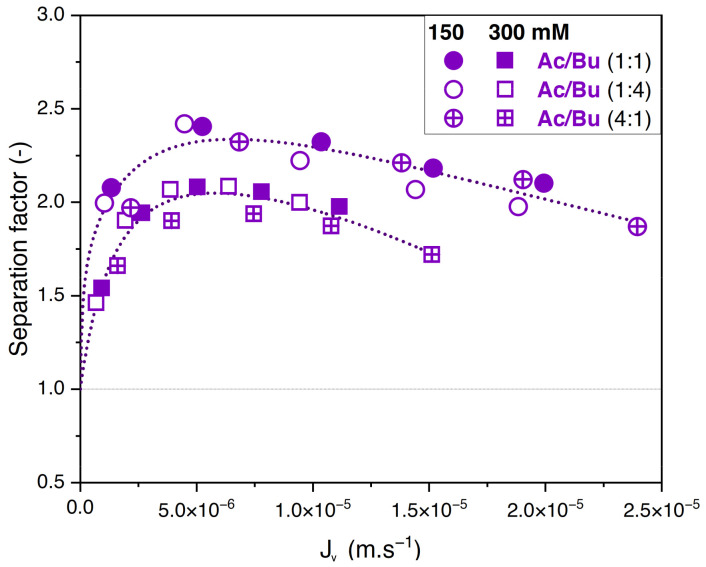
Separation factor of acetate/butyrate vs. filtration flux in binary solutions with different feed proportions at 150 and 300 mM using an NF270 membrane.

Whatever the total concentration, Figure 5A,B show no difference between the retention values obtained with the different feed proportions. The corresponding values of the separation factor of acetate/butyrate are plotted in Figure 6. As previously observed, lower separation factors are obtained for increasing feed concentrations, regardless of the feed proportion of solutes. Moreover, within the given conditions, the separation factor of acetate/butyrate is independent of the feed proportion. This is confirmed by a one-way ANOVA analysis of the data, which reveals that the means of the separation factor in each condition are not significantly different (*p* > 0.05).

**Figure 7:**
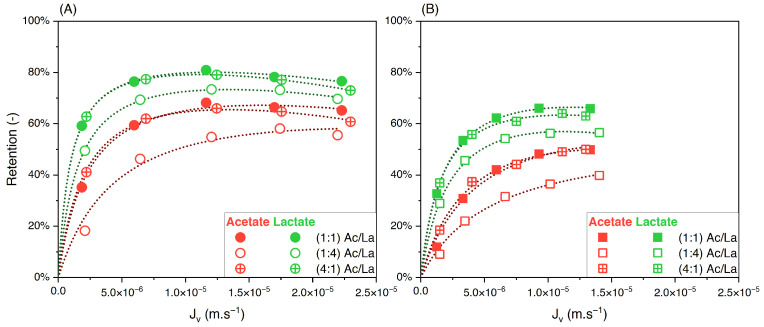
Retention of acetate and lactate vs. filtration flux—binary solutions of acetate/lactate with different feed proportions at (**A**) 150 and (**B**) 300 using an NF270 membrane.

The influence of varying the solute proportions on the feed was also investigated for the acetate/lactate binary solution. These results are plotted in Figure 7A,B.

**Figure 8:**
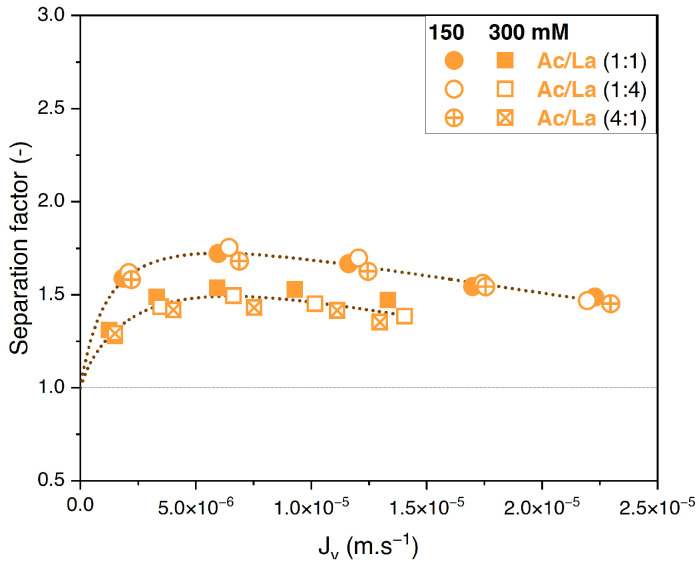
Separation factor of acetate/lactate vs. filtration flux in binary solutions with different feed proportions at 150 and 300 mM using an NF270 membrane.

In contrast to the acetate/butyrate binary, Figure 7A,B show that the retentions of acetate and lactate depend on the proportions of these solutes in the feed solution. Specifically, in the 1:4 (Ac/La) proportion, the retention of both solutes decreases compared to those in the 1:1 and 4:1 proportions. The corresponding values of the separation factor of acetate/lactate are plotted in Figure 8.

The separation factor of acetate/lactate remains independent of the feed proportions. This observation is further supported by statistical analysis, which shows that the means of the separation factor under varying conditions are not statistically different (*p* > 0.05). Therefore, while the retention of solutes in the acetate/lactate system is influenced by their feed proportions, this does not affect the separation factor. This is in agreement with the results obtained with the acetate/butyrate binary.

Experimental results obtained with butyrate/lactate solutions varying the solute proportions are given in detail in Supplementary Data. A statistical analysis was carried out about the influence of solute proportions on the separation factor. Although statistically significant variations were observed for higher lactate proportions at both 150 and 300 mM (*p* < 0.05), these differences remain very small. The separation factor averaged 1.16, ranging from 1.11 to 1.19 at 150 mM and averaged 1.19, ranging from 1.14 to 1.21 at 300 mM. These observations will be further contextualized in the discussion of results from ternary solutions.

Our findings are in accordance with previous conclusions of Zhu et al. (2020) [16] using the NF45 membrane and binary and ternary acetate, propionate, and butyrate solutions. They reported that feed concentration had no effect on the proportions of acetate and butyrate in the permeate. On the contrary, different permeate proportions were obtained for different feed proportions. However, based on the data given in this paper, one can calculate the separation factor between any two solutes and conclude that it is independent of their feed proportions.

In another study by Sjöman et al. (2007) [15], the impact of varying xylose to glucose mass ratios on the separation factor of monosaccharides was investigated using different nanofiltration membranes. At a given total monosaccharide concentration of 30% wt.%, three different xylose to glucose initial proportions (1:9), (1:1), and (9:1) were investigated with a Desal-5 DL membrane. The results demonstrated that increasing the glucose content in the feed led to lower xylose retention. However, when comparing the separation factor of xylose over glucose, calculated based on the xylose and glucose fluxes given in the paper, no influence of different initial proportions is pointed out. Moreover, the total solution concentration (between 2 and 30% weight) was found to have a minor effect as well.

In summary, for acetate/butyrate and acetate/lactate binaries, data show that while retentions can vary depending on the solute proportions (in the case of acetate/lactate), the separation factors remain constant for different feed proportions. In the case of butyrate/lactate solutions, despite a statistically significant difference in separation factor at higher lactate proportions, the variations remain very small. Overall, the different solute proportions in the feed do not affect the selectivity of nanofiltration expressed by the separation factor.

### 3.4. Ternary Solutions

**Figure 9:**
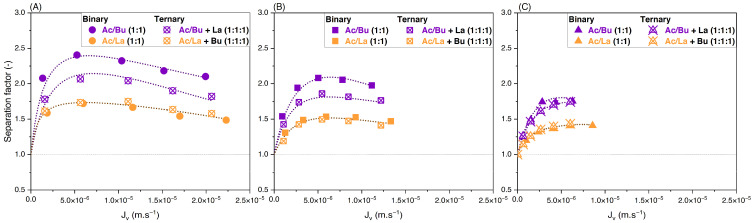
Separation factor vs. filtration flux—comparison between binary and ternary solutions at (**A**) 150 mM, (**B**) 300 mM, and (**C**) 500 mM using an NF270 membrane.

Experiments were then carried out with ternary solutions containing acetate, butyrate, and lactate at different feed concentrations and proportions. To ascertain the effect of the addition of a third solute on the separation factor of organic acids, data are compared between equimolar binary and ternary solutions. For example, results for acetate/butyrate in binary (1:1) are compared to their values in ternary with the addition of lactate (1:1:1). Values for retention of solutes in ternary solutions are presented in Supplementary Data. Figure 9 shows the separation factors of acetate/butyrate and acetate/lactate versus the filtration flux obtained with ternary solutions. 

One can observe that, for any concentration, the separation factor of acetate/lactate is not modified by the addition of the third solute, i.e., butyrate. On the contrary, the addition of lactate decreases the separation factor of acetate/butyrate. Moreover, the effect of feed concentration on the separation factor in ternary solution is similar to that observed for binaries, i.e., it decreases with increasing feed concentrations. The average separation factor of acetate/butyrate decreases from 1.87 to 1.78 and then to 1.69 at 150, 300, and 500 mM, respectively. Similarly, for acetate/lactate, the average separation factor decreases from 1.62 to 1.44 and then to 1.40 at the same concentrations in ternary.

**Figure 10:**
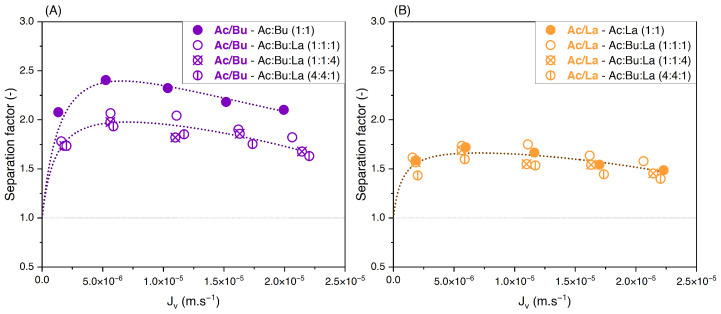
Separation factors of (**A**) acetate/butyrate and (**B**) acetate/lactate vs. filtration flux with different feed proportions at 150 mM using an NF270 membrane.

Given the influence of lactate on the separation factor of acetate/butyrate, experiments were carried out with varying proportions of acetate, butyrate, and lactate in ternary solutions. Since the impact of the presence of lactate seems to be more important at lower concentrations (Figure 9), the feed concentration was fixed at 150 mM. The results are given in Figure 10.

Figure 10A confirms that the separation factor of acetate/butyrate is significantly lower in the presence of lactate, whatever the proportions investigated. Moreover, the means for the separation factor in each condition are not significantly different (*p* > 0.05). In other words, while the presence of lactate diminishes the acetate/butyrate separation factor, this effect is not influenced by the proportion of lactate in the feed. This is in agreement with our previous observations in binary solutions, where the separation factor was also independent of feed proportions. In the case of acetate/lactate, the separation factor is affected neither by the presence of a third species, i.e., butyrate, nor by variations in feed proportions, as confirmed by statistical analysis (*p* > 0.05).

To summarize the influence of these conditions on the separation factor of lactate/butyrate, an ANOVA analysis was conducted for all conditions (experiments S10 to S15 given in Supplementary Data) to evaluate if there were statistically significant differences between the means. It was established that the means were different (*p* < 0.05). Subsequent to this, a Tukey test was performed, which demonstrated that the statistically significant difference originated from the experiments with the binary solution at initial concentrations of 150 and 300 mM, where the proportion of lactate was higher (S11). However, despite this statistical difference, from a practical perspective, the observed variations are minimal. The average value, considering all different conditions of concentration, proportion, and whether in binary or ternary solution, is 1.17 ± 0.05. This confirms that, in the case of the separation factor of lactate/butyrate, no significant influence of feed concentration or proportions is observed in both the binary and the ternary solutions. 

Few studies have focused on the separation factor of solutes in ternary solutions and how those compare to binary solutions. However, from the results reported by Zhu et al. (2020) [16], one can calculate the separation factor of couples of acetates, propionate, and butyrate and show that they are similar in binary or ternary and independent from the feed composition.

## 4. Conclusions

In this study, the effect of feed composition, specifically concentration and solute proportions, on the separation factor of organic acids during nanofiltration was investigated using an NF270 membrane. The retention and separation factors of acetate, butyrate, and lactate were investigated in single, binary, and ternary solutions. For single solutions, the retention order was butyrate>lactate>acetate, regardless of feed concentration. While they have the same molecular weight, the different retentions between butyrate and lactate show that other mechanisms than steric exclusion are involved.

Increasing the feed concentration decreases the separation factor for the binaries acetate/butyrate and acetate/lactate but does not affect the separation factor of the binary lactate/butyrate. This suggests that the electrostatic repulsion, which is more pronounced at lower feed concentrations, is favorable to the selectivity of nanofiltration as expressed by the separation factor. Furthermore, it was observed that for any couple of solutes, while the solute retentions can vary depending on solute proportions, the separation factor remains constant, whatever these proportions. 

In ternary solutions, it was observed that the separation factor remains independent of the solute proportions. The addition of lactate was found to decrease the separation factor of acetate/butyrate. On the contrary, the separation factor of acetate/lactate is identical with or without the addition of butyrate. The same is true for the separation factor of lactate/butyrate and the addition of acetate. 

In this study, experiments were conducted at a fixed pH 8, at which weak organic acids like acetate, butyrate, and lactate are fully dissociated and negatively charged. It was observed that the separation factor increased with increasing electrostatic repulsion, as shown with decreasing feed concentration. Since for organic acids electrostatic interactions also depend on the pH of the feed solution, the influence of the pH on the separation factor will be investigated. 

Further investigation could also be conducted in order to check if identical trends are observed with other nanofiltration membranes, to strengthen even more the generic character of the conclusions of this study.

Last but not least, experiments will be performed with more complex organic acid mixtures, like dark fermentation broths, in order to evaluate the robustness of the separation factor to characterize the selectivity of NF when used to treat complex mixtures. 

## Figures and Tables

**Table 1:** Chemical characteristics of acetate, butyrate, and lactate.

Name	Acetate	Butyrate	Lactate
Structure	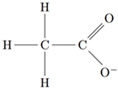	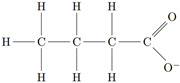	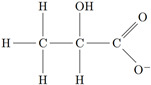
Molecular weight (g·mol^−1^)	59.04	87.09	89.07
pK_a_ (25 °C)	4.75	4.84	3.86

**Table 2:** Experimental set-up characteristics and operating conditions.

Maximum Volume	500 mL
Temperature	Room temperature (21–25 °C)
Pressure	4–20 bar
Compressed gas type	N_2_
Magnetic stirring speed	200 rpm
Active membrane area	50.3 cm^2^

**Table 3:** Composition of synthetic solutions used in experiments given in molar fraction.

	Single Solutions			Binary Solutions									Ternary Solutions		
	S1	S2	S3	S4	S5	S6	S7	S8	S9	S10	S11	S12	S13	S14	S15
Acetate (%)	100	-	-	50	20	80	50	20	80	-	-	-	33	17	44
Butyrate (%)	-	100	-	50	80	20	-	-	-	50	20	80	33	17	44
Lactate (%)	-	-	100	-	-	-	50	80	20	50	80	20	33	66	11

## Data Availability

The data presented in this study are available on request from the corresponding author.

## Supplementary Materials

The following supporting information can be downloaded at: https://www.mdpi.com/article/10.3390/membranes14080166/s1, Figure S1: Retention of butyrate and lactate vs. filtration flux—binary solutions of butyrate/lactate with different feed proportions at (A) 150 and (B) 300 using an NF270 membrane; Figure S2: Retention of acetate, butyrate, and lactate vs. filtration flux—comparison of retention in binary and ternary equimolar solutions over different feed concentrations; Figure S3: Separation factor La/Bu over all different conditions (concentration, proportion, binary, and ternary).

## Abbreviations

*J_v_*	Filtration flux (m·s^−1^)
*V_p_*	Volume of permeate (m^3^)
*t*	Time (s)
*S_m_*	Membrane surface (m^2^)
*L* *_p_*_0_	Membrane’s water permeability (m·s^−1^·Pa^−1^)
Δ*P*	Pressure difference (Pa)
*R*	Retention
*C*	Concentration (mol·m^−3^)
*SF*	Separation factor (-)
