# Supplementary material for: Influence of Feed Composition on the Separation Factor during Nanofiltration of Organic Acids
Source: Membranes (Basel). 2024 Jul 28;14(8):166. doi: 10.3390/membranes14080166 (PMC11356361; doi:10.3390/membranes14080166)

Supplementary data

Figure S1: Retention of butyrate and lactate vs. filtration flux - binary solutions of butyrate/lactate with different feed proportions at (A) 150, and (B) 300 using an NF270 membrane.

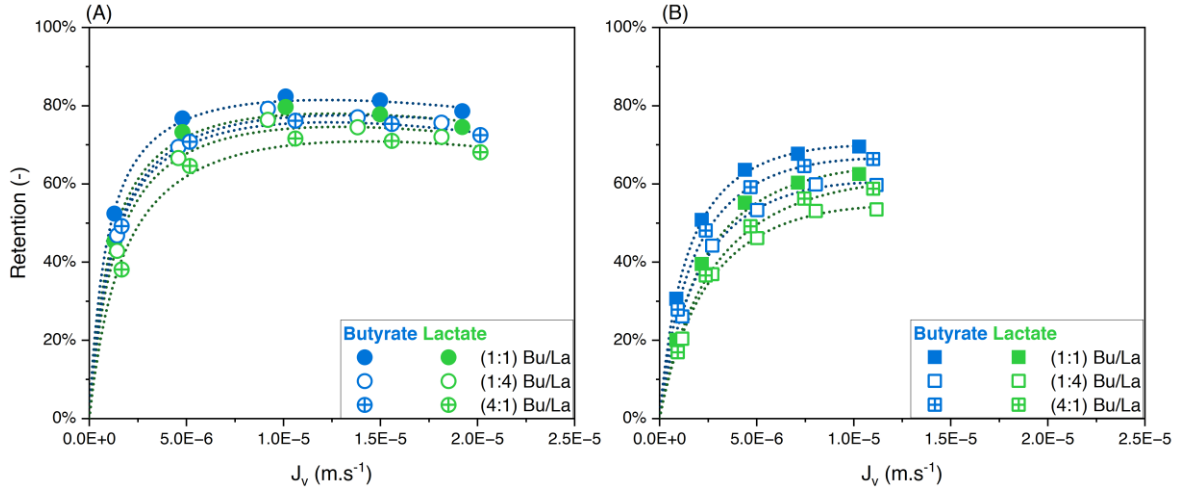

Figure S2: Retention of acetate, butyrate, and lactate vs. filtration flux – comparison of retention in binary and ternary equimolar solutions over different feed concentrations

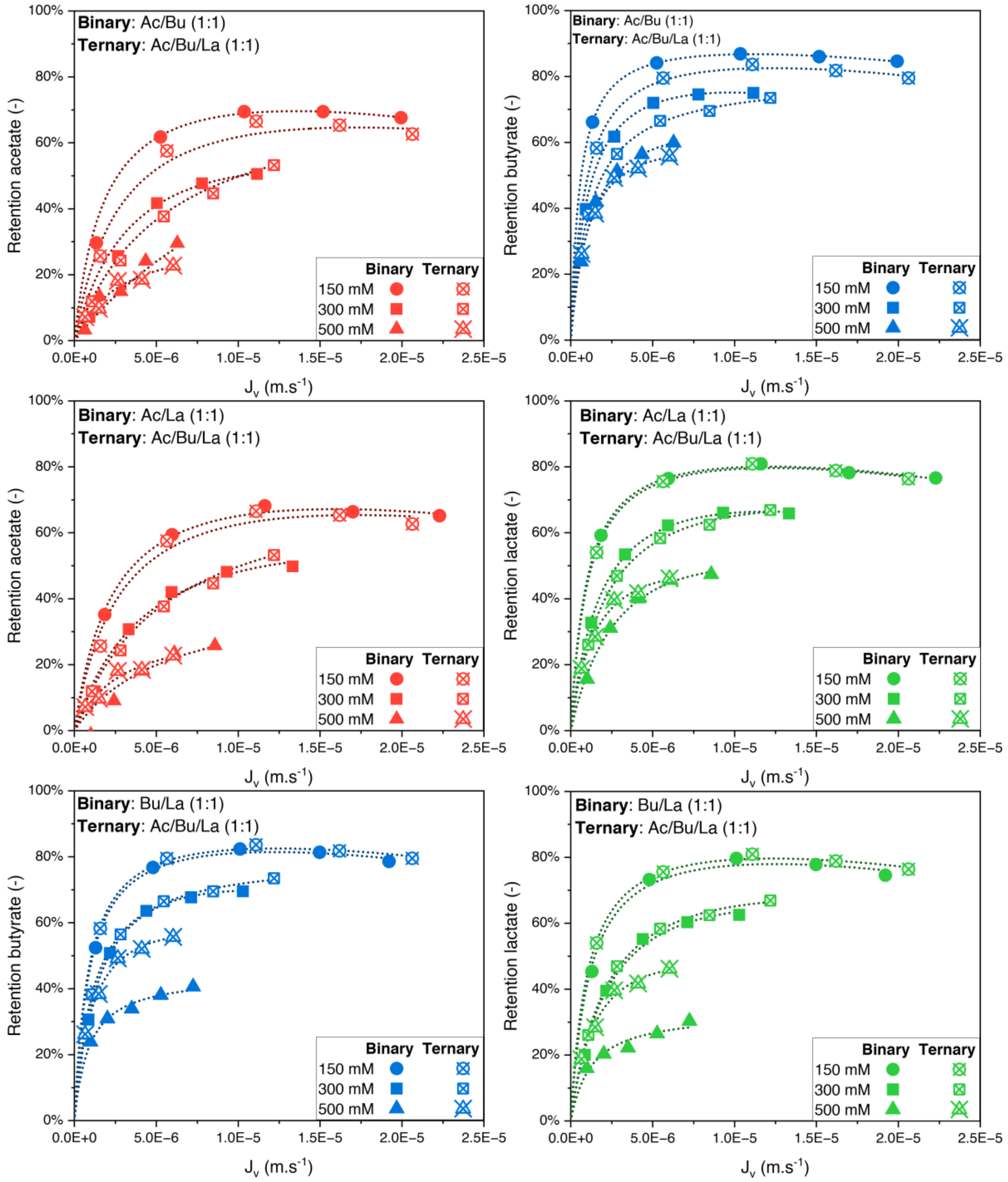

Figure S3: Separation factor La/Bu over all different conditions (concentration, proportion, binary, and ternary)

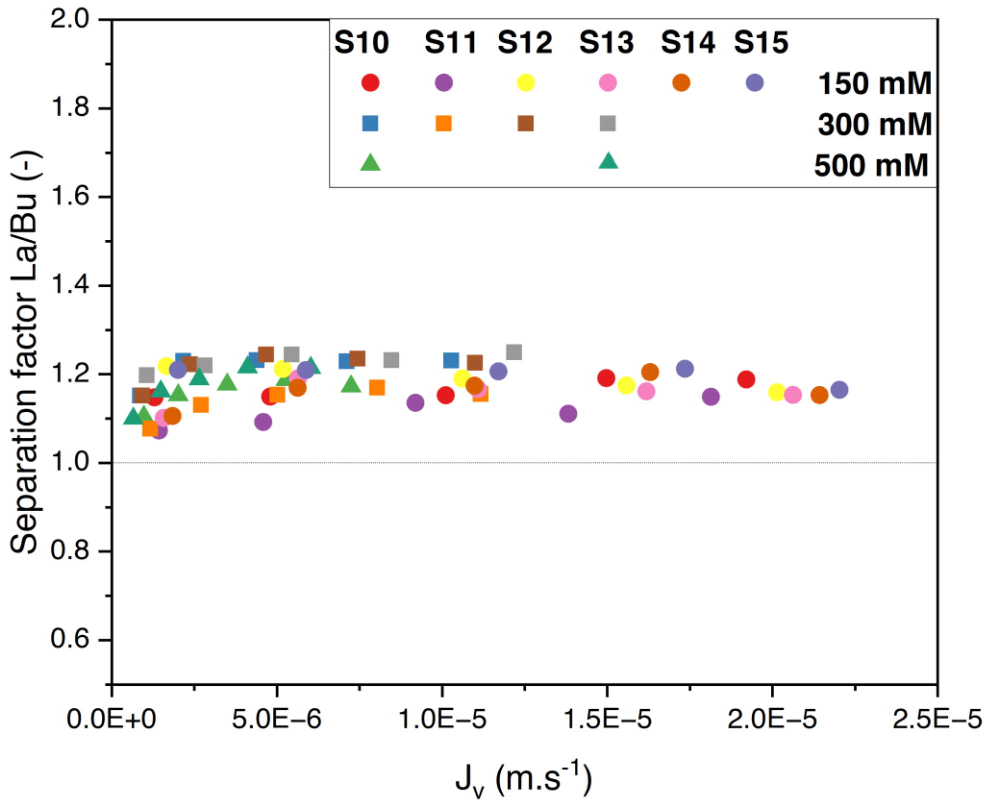

Supplement: Supplementary file 1 [file membranes-14-00166-s001.zip › membranes-3090337-supplementary.pdf]
